# Striving for better comunication - an audit

**DOI:** 10.1192/bjo.2021.86

**Published:** 2021-06-18

**Authors:** Eleanor Breen

**Affiliations:** NHS Lanarkshire

## Abstract

**Aims:**

The aim of this audit is to assess communication between the general and psychiatric hospital. This audit was prompted after a number of patients were transferred to Udston Hospital, a community hospital with two older adult acute mental health wards, with no written communication. This led to several significant issues including medication errors, ambiguity regarding patient escalation plans and uncertainty regarding what had been discussed with families.

**Method:**

Over the course of one month eight patients were identified who had been transferred from the acute site to Udston Hospital. Three were new admissions to Udston, four were returning after treatment for physical illness, and one returned following assessment in ED. Data were collected by examining paper and electronic notes, and analysed using Excel. The results of this audit were discussed at the local clinical governance meeting. A 2nd cycle was performed. Eight transfers were identified. Four were returning after an assessment in ED, two were new admissions to Udston and two were returning after treatment for physical illness.

**Result:**

Initial audit found that 38% of patients were transferred with their medical notes, 50% were transferred with no written documentation whatsoever, and none of the patients were transferred with a transfer letter. The second cycle found that 88% of patients had a transfer or discharge letter. 12% of patients came with no written documentation.

**Conclusion:**

The initial audit found significant deficiencies in communication. Highlighting the need for all patients to have a transfer letter at a local management meeting seems to have led to an improvement. However, differences between the samples in the 1st and 2nd audit cycle could be distorting the results. Further audits would be useful given the small sample size and due to the differences between the sample populations.

